# A Biomechanically Guided Multimodal Surgical Strategy for Cataract Surgery in an Eye with Extreme Corneal Thinning after Toxic Epidermal Necrolysis: A Case Report

**DOI:** 10.1159/cop/acaag020

**Published:** 2026-09-09

**Authors:** Hisaharu Suzuki, Katsuhiro Inoue

**Affiliations:** a Zengyo Suzuki Eye Clinic, Fujisawa, Japan; b Ofuna Inoue Eye Clinic, Kamakura, Japan

**Keywords:** Cataract surgery, Toxic epidermal necrolysis, Corneal thinning, Biomechanics, Intraocular pressure control

## Abstract

**Introduction:**

Cataract surgery in eyes with extreme corneal thinning secondary to toxic epidermal necrolysis (TEN) is technically challenging because of marked structural fragility and ocular surface instability. We report a multimodal surgical strategy that facilitated safe completion of cataract surgery in such a high-risk eye.

**Case Presentation:**

We report an 80-year-old woman with a history of TEN who presented with visually significant cataract in the right eye. She had severe localized corneal thinning with a minimum corneal thickness of 138 μm and a history of corneal perforation approximately 10 years earlier. Reliable keratometric measurements were not possible because of marked corneal irregularity, and anterior segment optical coherence tomography was used for structural assessment. Cataract surgery was performed using a combination of heads-up three-dimensional visualization with monochrome filtering, a transconjunctival single-plane sclerocorneal incision, dispersive ophthalmic viscosurgical device protection, external corneal support with a disposable diagnostic contact lens, and low intraoperative intraocular pressure (20–30 mm Hg) maintained with active fluidics. The procedure was completed without intraoperative complications, and visual acuity improved from 0.04 to 0.10 (decimal) at 1 week postoperatively, with a stable corneal appearance. According to follow-up information obtained from the referring ophthalmologist, a delayed corneal perforation subsequently occurred at the site of the preexisting thinning approximately 2 months after surgery and was managed with a therapeutic soft contact lens; visual acuity had further improved to 0.30 at last available follow-up.

**Conclusion:**

A biomechanically guided multimodal surgical strategy combining enhanced visualization, ocular surface protection, external corneal support, a transconjunctival single-plane sclerocorneal incision, and controlled low intraoperative intraocular pressure may facilitate safe completion of cataract surgery in carefully selected eyes with extreme corneal thinning. This case highlights the potential value of minimizing and redistributing both externally and internally generated mechanical stress as a practical surgical framework for such eyes, although the underlying corneal structural fragility itself may not be resolved by cataract surgery alone.

## Introduction

Cataract surgery in eyes with severe ocular surface disease is technically challenging because chronic inflammation, corneal irregularity, tear film instability, and structural corneal abnormalities can substantially impair surgical visualization and increase the risk of intraoperative complications [1]. Among these conditions, chronic ocular sequelae of Stevens-Johnson syndrome (SJS) and toxic epidermal necrolysis (TEN) represent some of the most demanding surgical situations owing to severe ocular surface damage, cicatricial changes, limbal stem cell dysfunction, and progressive corneal thinning [2–5].

Although previous studies have demonstrated that cataract surgery can be successfully performed in carefully selected eyes with chronic SJS/TEN using individualized surgical modifications [2, 3], no standardized surgical strategy has been established for eyes with extreme corneal thinning in which profound biomechanical fragility substantially increases the risk of intraoperative tissue damage. Furthermore, the literature has largely focused on management of ocular surface disease itself rather than on surgical strategies specifically designed to minimize mechanical stress on structurally compromised corneas.

We report cataract surgery in an eye with extreme corneal thinning (minimum corneal thickness, 138 μm) secondary to TEN using a multimodal surgical strategy that combined enhanced visualization, external corneal support, ocular surface protection, a transconjunctival single-plane sclerocorneal incision, and strict intraoperative intraocular pressure control. Rather than describing an isolated surgical modification, this report highlights a comprehensive strategy aimed at minimizing and redistributing mechanical stress on an extremely fragile cornea.

## Case Presentation

An 80-year-old woman was referred to our clinic because of progressive visual impairment in her right eye caused by cataract. She had developed TEN 11 years previously, followed by chronic ocular surface disease with severe localized corneal thinning. According to the referral records, the right eye had sustained a corneal perforation approximately 10 years before presentation, which subsequently healed. Four months later, she developed a corneal ulcer complicated by endophthalmitis. Although the infection resolved, marked localized corneal thinning remained.

She had undergone punctal occlusion for severe dry eye and was receiving long-term topical treatment with preservative-free hyaluronic acid, diquafosol sodium, rebamipide, and fluorometholone. Best-corrected visual acuity was 0.04 (decimal) in the right eye and 0.01 in the left eye. Intraocular pressure was 13 mm Hg in both eyes.

Slit-lamp examination revealed marked localized corneal thinning with stromal scarring outside the visual axis (Fig. 1a, c). Anterior segment optical coherence tomography demonstrated a minimum corneal thickness of 138 μm (Fig. 1b). Reliable keratometric measurements could not be obtained because of severe corneal irregularity. There was no corneal neovascularization or other clinical evidence of limbal stem cell deficiency. Fundus examination showed no remarkable posterior segment abnormalities that could explain the visual impairment.

**Fig. 1.**
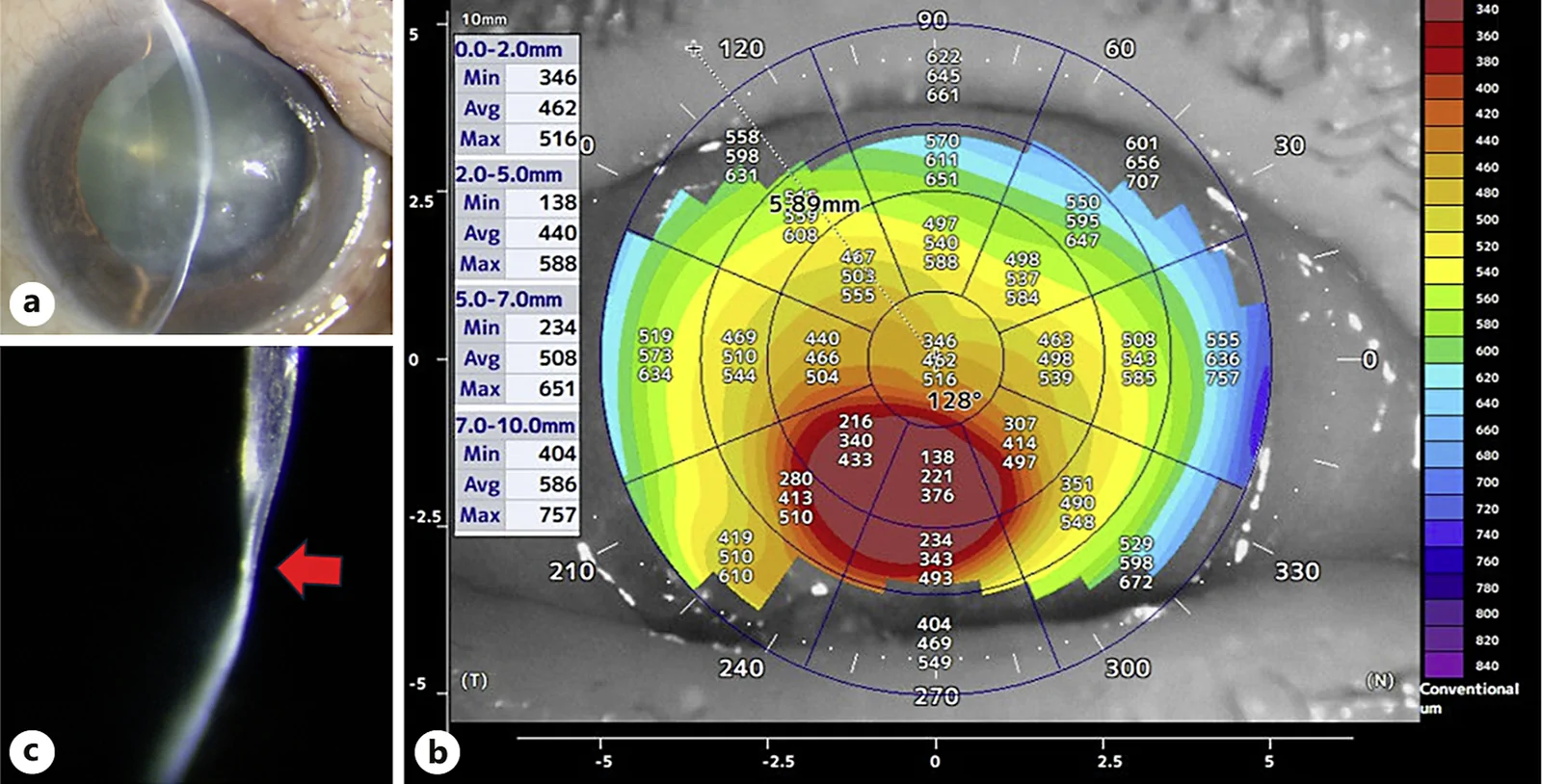
Preoperative anterior segment findings. **a** Slit-lamp photograph showing the overall anterior segment appearance of the right eye. **b** Anterior segment optical coherence tomography pachymetry map demonstrating localized corneal thinning with a minimal corneal thickness of 138 µm. **c** Slit-lamp photograph (optical section) highlighting the area of maximal corneal thinning (arrow).

Because of the patient’s strong desire for visual improvement and the visually significant cataract, cataract surgery was planned. Given the extreme corneal thinning and previous history of corneal perforation, a surgical strategy was designed to minimize mechanical stress on the fragile cornea throughout the procedure.

### Surgical Technique

To minimize mechanical stress on the extremely thinned cornea throughout surgery, a multimodal surgical strategy was adopted. Heads-up three-dimensional visualization with a monochrome filter (NGENUITY^®^, Alcon Laboratories, Inc., Fort Worth, TX, USA) was used to enhance contrast through the irregular cornea and facilitate precise continuous curvilinear capsulorhexis (Fig. 2a). Anterior capsule visibility was judged sufficient with this visualization strategy alone, and trypan blue staining was therefore not used; in eyes with poorer capsular visibility than the present case, trypan blue may serve as a useful complementary aid to facilitate capsulorhexis.

**Fig. 2.**
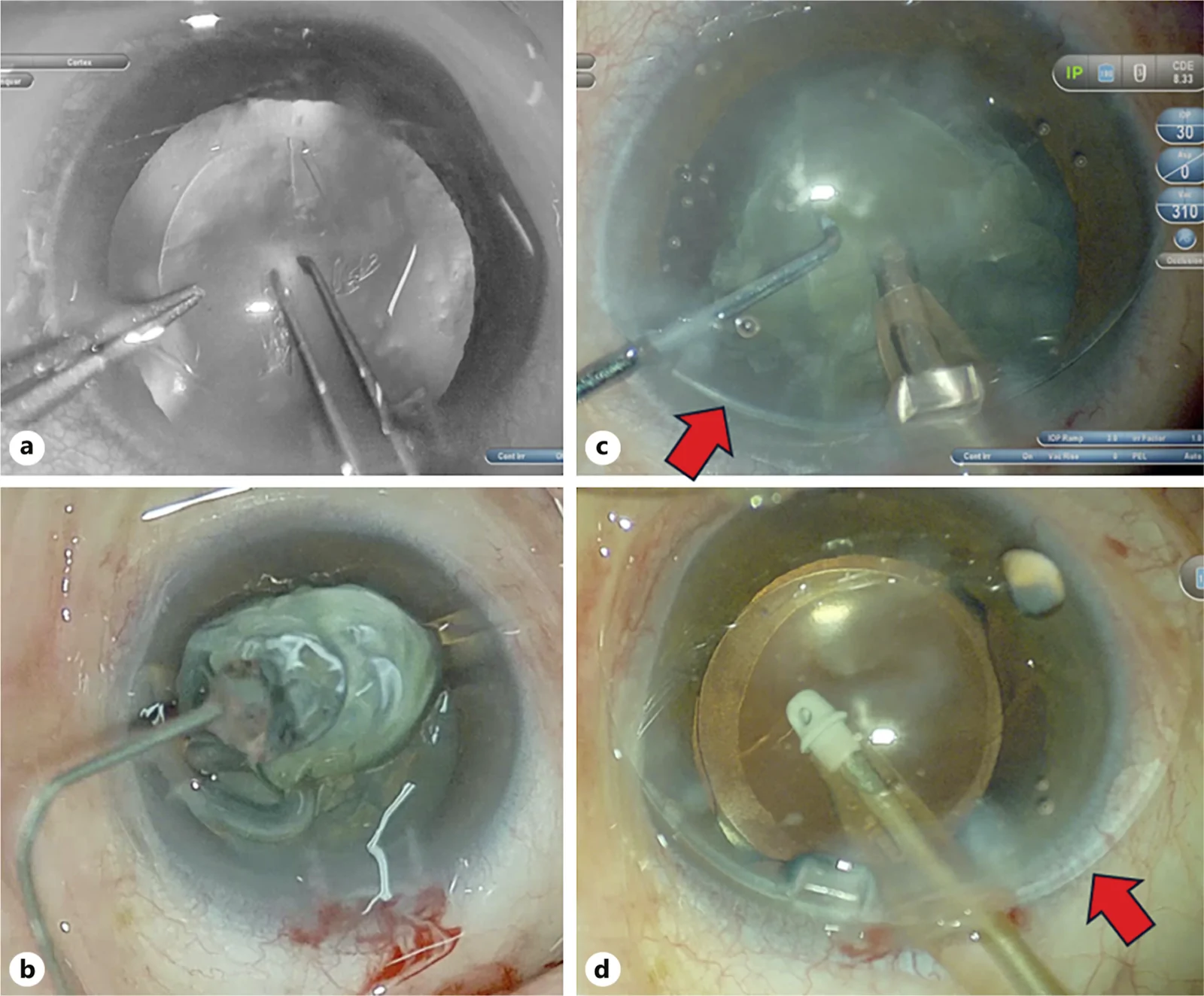
Intraoperative protective surgical strategy. **a** Continuous curvilinear capsulorhexis performed using heads-up surgery with a monochrome filter to enhance visualization. **b** Application of a dispersive ophthalmic viscosurgical device over the corneal surface. **c** Phacoemulsification performed with the disposable diagnostic contact lens (arrow) placed on the cornea to provide external support. **d** The disposable diagnostic contact lens (arrow) was maintained in place until completion of intraocular lens implantation.

Because additional trauma to the fragile cornea was considered undesirable, a transconjunctival single-plane sclerocorneal incision was created instead of a clear corneal incision. Unlike a conventional sclerocorneal tunnel incision, which is typically constructed after a wide conjunctival peritomy with the scleral and corneal components fashioned as separate, stepped dissections, this technique creates the conjunctival, scleral, and peripheral corneal components of the wound in a single continuous plane through two small relaxing conjunctival incisions, an approach previously described to minimize conjunctival trauma while retaining the wound-security advantages of a scleral incision [6]. We selected this technique, rather than a conventional sclerocorneal tunnel incision, specifically to minimize conjunctival and scleral manipulation in this eye with severely compromised ocular surface integrity. A dispersive ophthalmic viscosurgical device (Viscoat^®^, Alcon Laboratories, Inc.) was injected into the anterior chamber and also applied to the corneal surface to protect the ocular surface and maintain a stable optical interface throughout surgery (Fig. 2b).

To reduce focal mechanical stress on the area of extreme corneal thinning, a rigid disposable diagnostic contact lens (HHV Disposable Contact Lens, Type Zd; HOYA Corporation, Tokyo, Japan), originally designed for vitreoretinal surgery, was placed over the cornea throughout phacoemulsification (Fig. 2c). Rather than being used solely to improve visualization, the lens was intended to distribute externally applied forces over a wider corneal surface, thereby reducing localized stress on the markedly thinned region while simultaneously providing a stable optical interface.

Phacoemulsification was performed using the Centurion^®^ Vision System equipped with an Active Sentry™ handpiece (Alcon Laboratories, Inc.). Because sudden increases in intraocular pressure could preferentially transmit stress to the structurally weakest area of the cornea, a deliberately low target intraoperative intraocular pressure of 20–30 mm Hg was maintained using the Active Fluidics™ system to minimize pressure fluctuations during surgery. The target IOP was set at 20 mm Hg during grooving and at 20–30 mm Hg during nucleus removal. During grooving, aspiration flow rate was 21 mL/min and vacuum was 80 mm Hg; during nucleus removal, aspiration flow rate was set at 18–22 mL/min under linear control and vacuum was increased to 310 mm Hg. Torsional ultrasound amplitude was maintained at 40% throughout the procedure. The nucleus was graded as Emery-Little grade 2.

Phacoemulsification and implantation of a posterior chamber intraocular lens (XC-1; HOYA Corporation, Tokyo, Japan) were completed uneventfully without intraoperative complications (Fig. 2d), with a cumulative dissipated energy of 10.76. Key intraoperative steps of this protective strategy are shown in Online supplementary Video 1 (for all online suppl. material, see https://doi.org/10.1159/cop/acaag020).

### Outcomes

The surgical procedure itself was completed without intraoperative complications. At 1 week postoperatively, slit-lamp examination demonstrated a stable corneal appearance without progression of the preexisting localized thinning (Fig. 3). Best-corrected visual acuity improved from 0.04 preoperatively to 0.10 (decimal), and no postoperative inflammation or wound-related complications were observed.

**Fig. 3.**
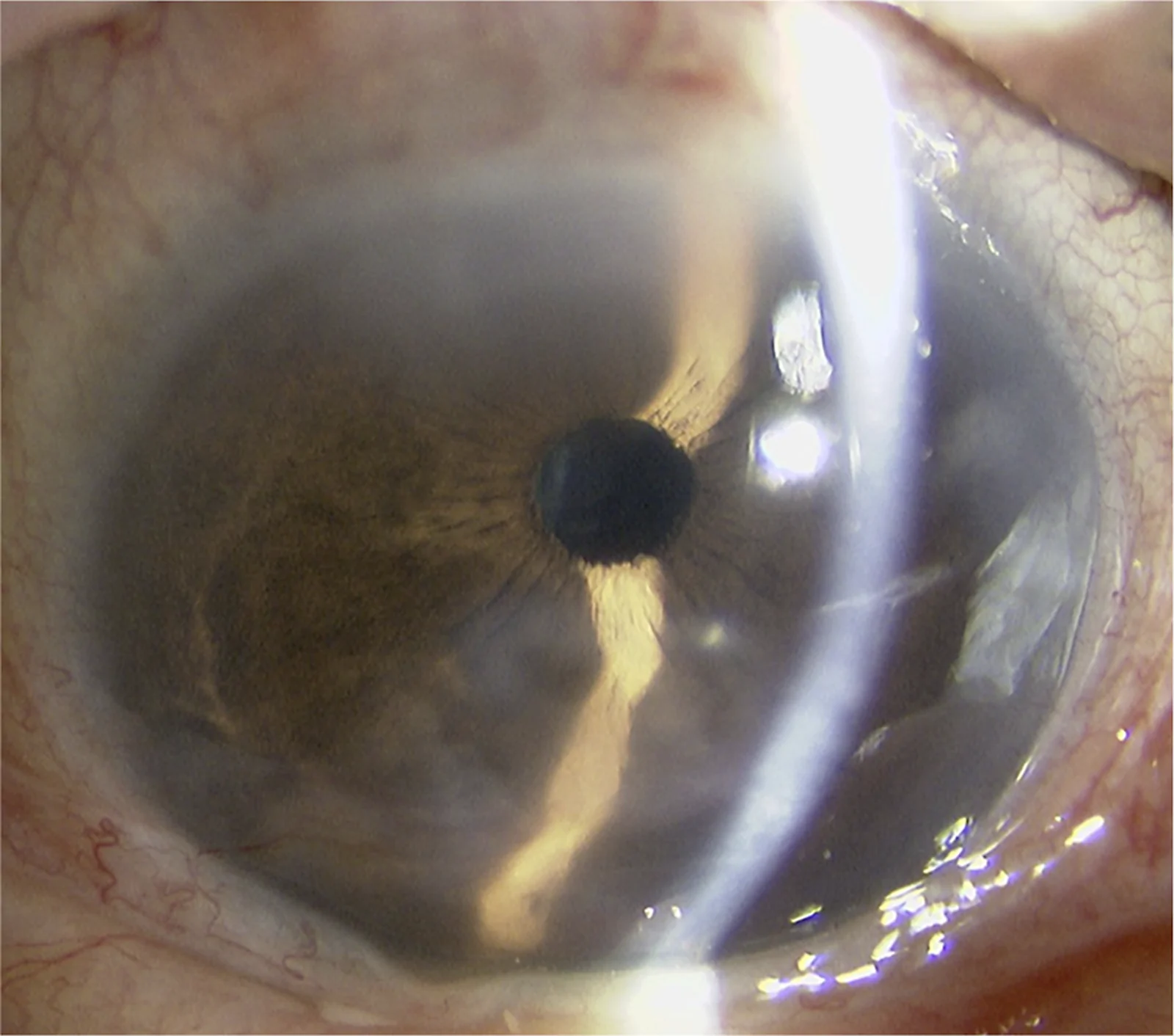
Postoperative anterior segment findings.

The patient was subsequently referred back to the referring ophthalmologist according to the routine postoperative follow-up protocol because of advanced age and limited ability to continue follow-up at our institution. According to information obtained from the referring ophthalmologist, localized perforation developed in the previously thinned corneal area approximately 2 months after surgery and was successfully managed with a therapeutic soft contact lens. At the last follow-up available to our institution, best-corrected visual acuity had further improved to 0.30 (decimal). No additional long-term follow-up data were available. The delayed perforation occurred at the site of preexisting extreme corneal thinning rather than at the surgical incision. This delayed perforation indicates that the underlying corneal structural fragility was not resolved by cataract surgery, even though the surgical procedure itself and the early postoperative course were uneventful.

## Discussion

Extreme corneal thinning presents a unique challenge during cataract surgery, as even routine manipulation and transient IOP fluctuations may damage structurally compromised tissue. Previous studies have shown that cataract surgery can be performed successfully in selected eyes with chronic SJS/TEN through careful preoperative optimization and individualized planning [2, 3], but these reports focused on ocular surface management rather than on strategies to reduce mechanical stress in profoundly thinned corneas. In the present case, the minimum corneal thickness was only 138 μm and the eye had a previous history of perforation. We therefore adopted a multimodal strategy aimed at minimizing and redistributing mechanical stress throughout the procedure, rather than relying on any single modification.

The rationale was based on the biomechanical behavior of an extremely thinned cornea; reduced tissue thickness and stiffness alter stress distribution and increase susceptibility to localized deformation and structural failure under mechanical loading [7, 8]. Although these principles have not been studied specifically in TEN, they are likely applicable to any cornea with profound structural fragility. We therefore combined multiple complementary modifications to reduce both externally applied forces (e.g., surgical manipulation and corneal contact) and internally generated forces (e.g., transient IOP fluctuations during phacoemulsification), redistributing stress away from the thinned cornea toward relatively preserved tissue rather than relying on any single maneuver. This general principle is likely broadly applicable regardless of etiology; the specific combination of techniques used here, however, was tailored to this patient and should not be regarded as a fixed protocol.

The biomechanical concept was translated into surgical practice through four complementary modifications, each intended to reduce a different source of external mechanical stress; the transconjunctival single-plane sclerocorneal incision, previously validated as reducing conjunctival trauma while retaining the wound-security advantages of a scleral incision [6], avoided direct manipulation of the thinned cornea, consistent with modifications reported for other severe corneal disorders such as peripheral ulcerative keratitis [9, 10]; the disposable rigid contact lens was intended to distribute externally applied forces over a wider corneal surface, rather than being used solely to improve visualization as previously described for irregular corneas [11]; dispersive ophthalmic viscosurgical device coating protected the ocular surface and reduced repeated epithelial manipulation [12]; and heads-up three-dimensional visualization minimized the illumination and manipulation required to complete key intraocular steps [13]. Full technical details of each modification are provided in the Surgical Technique section above and are not repeated here. Although the stress-redistribution effect of the contact lens is biomechanically plausible, it remains theoretical and was not directly measured in this eye; the present case cannot determine its contribution, and further investigation is required.

Maintaining intraoperative anterior chamber stability was considered equally important, because sudden IOP fluctuations may preferentially transmit stress to the weakest region of an extremely thinned cornea. In our previous experimental work, the Active Sentry handpiece reduced anterior chamber depth fluctuations and surge during occlusion break compared with a conventional system [14], and a subsequent randomized trial showed that Active Sentry combined with Active Fluidics enables safe phacoemulsification at lower target IOP [15]. Based on these findings, we selected a deliberately low target IOP of 20–30 mm Hg throughout surgery; although its protective effect cannot be confirmed from a single case, this is consistent with our previous work showing that improved intraoperative pressure control reduces abrupt pressure-related change and posterior capsule displacement [16].

Alternative surgical strategies for eyes with severe corneal thinning after TEN also merit consideration. In eyes with evident limbal stem cell deficiency or diffuse corneal compromise, limbal stem cell transplantation combined with keratoplasty may provide both tectonic and visual benefit. In the present case, slit-lamp examination showed no corneal neovascularization or other evidence of limbal stem cell deficiency, and the thinning was localized rather than diffuse; we therefore did not pursue keratoplasty, given the substantially greater invasiveness, surgical risk, and requirement for long-term immunosuppression that keratoplasty would entail in an eye with chronic TEN-related ocular surface disease, together with the patient’s advanced age. We consider limbal stem cell transplantation and keratoplasty an important alternative to weigh in eyes with more extensive limbal or diffuse corneal involvement than the present case.

Several limitations should be acknowledged. First, this is a single case, and the individual contribution of each component of the multimodal strategy, including the contact lens, whose role is discussed above, cannot be determined. Second, the proposed concept of mechanical stress redistribution remains theoretical, as direct biomechanical measurements or finite element analyses were not performed. Third, long-term postoperative observation was limited because the patient returned to the referring ophthalmologist after the early postoperative period; although the delayed corneal perforation occurred at the site of the preexisting extreme thinning rather than at the surgical incision, it remains uncertain whether this event reflected the natural progression of the underlying ocular surface disease or was influenced by the surgical intervention. Fourth, preoperative documentation was necessarily focused on surgical planning given the patient’s advanced age and limited capacity for extended examination; additional imaging such as scleral scatter illumination or retroillumination was not obtained, and objective ocular surface findings such as fluorescein staining, tear film breakup time, and Schirmer testing were not recorded, so only the clinical diagnosis of severe dry eye disease and its treatment regimen can be reported. Fifth, specular microscopy was attempted preoperatively and postoperatively, but marked corneal surface irregularity precluded reliable image acquisition, and the resulting endothelial cell density values were biologically implausible and are therefore not reported.

Nevertheless, this case demonstrates that an integrated biomechanical strategy may facilitate safe completion of cataract surgery in an eye with profound corneal fragility, even though the underlying fragility itself was not resolved, as illustrated by the delayed perforation. This general principle may warrant evaluation in other causes of severe corneal thinning, including peripheral ulcerative keratitis, neurotrophic keratopathy, autoimmune corneal melt, and advanced ocular surface disease, although further clinical experience is required.

## Conclusion

This case demonstrates that safe completion of cataract surgery in an eye with extreme corneal thinning requires more than any single technical modification. A comprehensive strategy – combining enhanced visualization, ocular surface protection, external corneal support, a transconjunctival single-plane sclerocorneal incision, and controlled low intraoperative IOP – may help redistribute mechanical stress away from the weakest region of the cornea during surgery. This concept addresses the safe completion of surgery itself, not the separate, unresolved problem of the eye’s long-term structural fragility, which persisted in this case despite an uneventful operation. Although further clinical experience is needed, this framework may be a practical starting point for cataract surgery in eyes with profound corneal structural fragility, regardless of etiology.

## Acknowledgments

The authors thank Editage (https://www.editage.com) for English language editing. Artificial intelligence (ChatGPT, OpenAI, and Claude, Anthropic) was used for language editing and translation during manuscript preparation; the authors reviewed and approved all AI-assisted content and take full responsibility for the final manuscript.

## Statement of Ethics

This study was reviewed by the Zengyo Suzuki Institutional Review Board (Approval No. 21000029), which determined that formal ethical review was not required for this retrospective single-case report. The study was conducted in accordance with the tenets of the Declaration of Helsinki. Written informed consent was obtained from the patient for the surgical procedure as well as for publication of this case report and the accompanying images. This case report was prepared in accordance with the CARE reporting guidelines, and the completed CARE checklist is provided as supplementary material [17].

## Conflict of Interest Statement

Hisaharu Suzuki reports research funding from Alcon and serving on its advisory boards. He has also served as a consultant for NIDEK and CREWT. These relationships are unrelated to the present study. Katsuhiro Inoue has no conflicts of interest to declare.

## Funding Sources

The authors received no financial support for this work.

## Author Contributions

Hisaharu Suzuki: conceptualization, data curation, formal analysis, investigation, methodology, project administration, resources, validation, visualization, and writing – original draft, review, and editing. Katsuhiro Inoue: data curation, project administration, resources, validation, and visualization.

## Data Availability

The data supporting the findings of this case report are not publicly available because they contain information that could compromise patient privacy but are available from the corresponding author upon reasonable request.
